# Daidzein Protects Caco-2 Cells against Lipopolysaccharide-Induced Intestinal Epithelial Barrier Injury by Suppressing PI3K/AKT and P38 Pathways

**DOI:** 10.3390/molecules27248928

**Published:** 2022-12-15

**Authors:** Baoping Zhang, Xiaohan Wei, Mengze Ding, Zhenye Luo, Xiaomei Tan, Zezhong Zheng

**Affiliations:** 1 School of Traditional Chinese Medicine, Southern Medical University, Guangzhou 510515, China; 2 Guangzhou Provincial Key Laboratory of Chinese Medicine Pharmaceutics, Southern Medical University, Guangzhou 510515, China; 3 Guangdong Provincial Engineering Laboratory of Chinese Medicine Preparation Technology, Guangzhou 510515, China; 4 College of Veterinary Medicine, South China Agricultural University, Guangzhou 510642, China

**Keywords:** daidzein, Caco-2 cells, intestinal epithelial barrier injury, AQP3, PI3K/AKT, P38

## Abstract

The intestinal epithelium provides an important barrier against bacterial endotoxin translocation, which can regulate the absorption of water and ions. The disruption of epithelial barrier function can result in water transport and tight junction damage, or further cause diarrhea. Therefore, reducing intestinal epithelial barrier injury plays an important role in diarrhea. Inflammatory response is an important cause of intestinal barrier defects. Daidzein improving the barrier integrity has been reported, but the effect on tight junction proteins and aquaporins is not well-described yet, and the underlying mechanism remains indistinct in the human intestinal epithelium. This study aimed to investigate the effects and mechanisms of daidzein on intestinal epithelial barrier injury induced by LPS, and a barrier injury model induced by LPS was established with human colorectal epithelial adenocarcinoma cell line Caco-2 cells. We found that daidzein protected the integrity of Caco-2 cell monolayers, reversed LPS-induced downregulation of ZO-1, occludin, claudin-1, and AQP3 expression, maintained intercellular junction of ZO-1, and suppressed NF-κB and the expression of inflammatory factors (TNF-α, IL-6). Furthermore, we found that daidzein suppressed the phosphorylation of the PI3K/AKT and P38 pathway-related proteins and the level of the related genes, and the PI3K/AKT and P38 pathway inhibitors increased ZO-1, occludin, claudin-1, and AQP3 expression. The study showed that daidzein could resist LPS-induced intestinal epithelial barrier injury, and the mechanism is related to suppressing the PI3K/AKT and P38 pathways. Therefore, daidzein could be a candidate as a dietary supplementation or drug to prevent or cure diarrhea.

## 1. Introduction

Infectious diarrhea is the second most common cause of illness and death in infants, and is an inescapable public health issue [1,2]. Pathophysiological mechanisms of infectious diarrhea include injury in epithelial tight junctions, a reduction in water transport proteins, and imbalance in the intestinal flora [3,4]. Pathogens colonizing the gut lumen produce toxins, which then stimulate the secretion of chemokines and pro-inflammatory cytokines, thus aggravating the colonic epithelial barrier disruption [5]. The resulting water transport and tight junction damage will further cause diarrhea. However, the main treatment of diarrhea currently includes fluid replacement such as oral rehydration and intravenous rehydration, which have finite curative effects [6]. Therefore, strategies to reduce intestinal barrier damage plays an important role in diarrheal therapy such as improving epithelial tight junctions and water transport [3].

The intestinal epithelial barrier is lined with transcellular and paracellular pathways, which together regulate the absorption of water and ions to maintain intestinal homeostasis [7]. Tight junction (TJ) proteins are the primary component in the paracellular pathway. TJ proteins can be divided into cytoplasmic adaptor proteins, zonula occludens (ZO) proteins, and the tetra-spanning membrane proteins, occludin and claudins [8]. The changes in TJ proteins cause epithelial barrier dysfunction, which could lead to diarrhea [9]. Aquaporins (AQPs), water-channel membrane proteins, are physiologically crucial for water transport homeostasis in the human digestive system including AQP1, AQP2, AQP3, AQP8, and so on [10]. Among them, AQP3 is regarded as a significant element in controlling fecal water content, as such, changing the expression of AQP3 can lead to clinical symptoms of diarrhea or constipation [11,12].

Lipopolysaccharide (LPS) is the principal composition of the outer membrane of Gram-negative bacteria cell walls. Studies have shown that a systemic inflammatory response was caused by LPS derived from *Escherichia coli* and *Shigella flexneri* [13]. Furthermore, LPS could induce TJ protein injury by triggering inflammatory signal cascades, and activating inflammatory mediators (e.g., tumor necrosis factor-α (TNF-α) and interleukin-6 (IL-6)) [14,15]. An in vitro study using the human colonic epithelial cell line HT-29 showed that AQP3 expression was decreased by LPS through the P38 and JNK signaling pathway [16].

**Figure 1:**
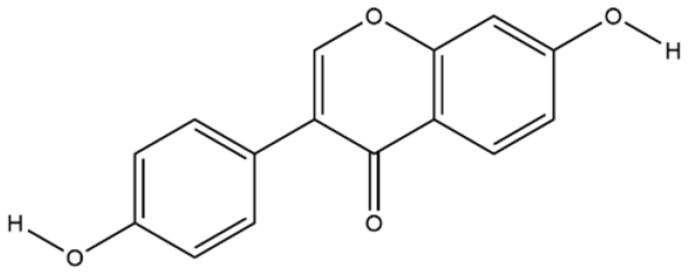
Structure of daidzein.

Daidzein (4′,7-dihydroxyisoflavone, Figure 1) is an isoflavone extracted from soy products [17] and the roots of *Pueraria lobata* (Gegen in China) [18]. It has been reported that daidzein inhibited dextran sodium sulfate (DSS)-induced murine colitis and the activity of LPS-induced IκB protein phosphorylation [19]. According to a previous report, daidzein can increase the capacity of anti-oxidative and the gene expression of TJ proteins in the intestinal of turbot [20,21], while the effect of daidzein on TJ and AQP3 proteins in the human intestinal epithelium and its potential mechanisms remains unconfirmed.

The human colon Caco-2 cell lines could be utilized to study the damage of TJ and AQP proteins in the intestinal epithelial barrier [21,22]. The aim of the study was to confirm the beneficial effects of daidzein on the integrity of the intestinal epithelial barrier. We examined the possible impacts and mechanism of daidzein on the cell monolayer permeability as well as the expression of intestinal epithelial proteins in Caco-2 cell inflammatory damage irritated by LPS.

## 2. Results

### 2.1. Cytotoxicity of LPS, Daidzein, LY294002, and SB203580

**Figure 2:**
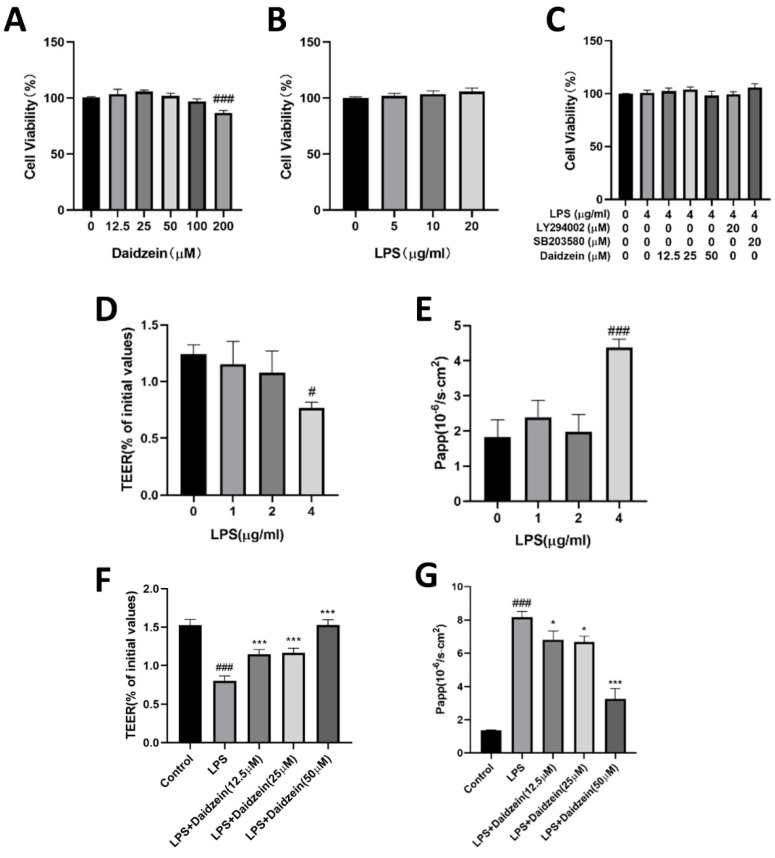
Daidzein improved lipopolysaccharide (LPS)-induced integrity disruption in Caco-2 cell monolayers. (**A**) Cytotoxicity of LPS for 24 h. (**B**) Cytotoxicity of daidzein for 24 h. (**C**) Cytotoxicity of different administration treatments for 24 h. (**D**,**E**) TEER and FD4 flux of different concentrations of LPS. (**F**,**G**) TEER and FD4 flux of different concentrations of daidzein (mean ± SD, *n* = 3), * *p* < 0.05, and *** *p* < 0.001 versus LPS-treated alone. # *p* < 0.05, ### *p* < 0.001 versus 0 dose treatment or control group.

The effect of LPS, daidzein, LY294002, and SB203580 on the cytotoxicity of Caco-2 cells was performed by CCK-8 Kits. Incubation with LPS (5–20 μg/mL) or daidzein (12.5–200 μM), or daidzein (12.5–50 μM) plus LPS (4 μg/mL), or LY294002 (20 μM) plus LPS (4 μg/mL), or SB203580 (20 μM) plus LPS (4 μg/mL) for 24 h had no statistically significant differences on the Caco-2 cell viability (*p* > 0.05), while 200 μM daidzein incubation reduced their viability (*p* < 0.001) (Figure 2A–C).

### 2.2. Daidzein Improved LPS-Induced Integrity Disruption in Caco-2 Cell Monolayer

The TEER and flux of the FITC-FD4 assays were performed to investigate whether daidzein provided protective effects against LPS-induced integrity disruption. Compared with the control group, a significant decrease in TEER (*p* < 0.05) and an increase in FITC-FD4 flux (*p* < 0.05) were observed after incubation with 4 μg/mL LPS for 24 h (Figure 2D,E). However, both the decreased TEER and the increased FITC-FD4 flux induced by 4 μg/mL LPS were dramatically improved (*p* < 0.05) by daidzein (12.5, 25, or 50 μM) treatment (Figure 2F,G), which confirmed that daidzein had a protective effect on the intestinal epithelial barrier of the Caco-2 monolayer.

### 2.3. Daidzein Increased LPS-Induced Downregulated Expression of TJ and AQP3 Proteins and mRNA

**Figure 3:**
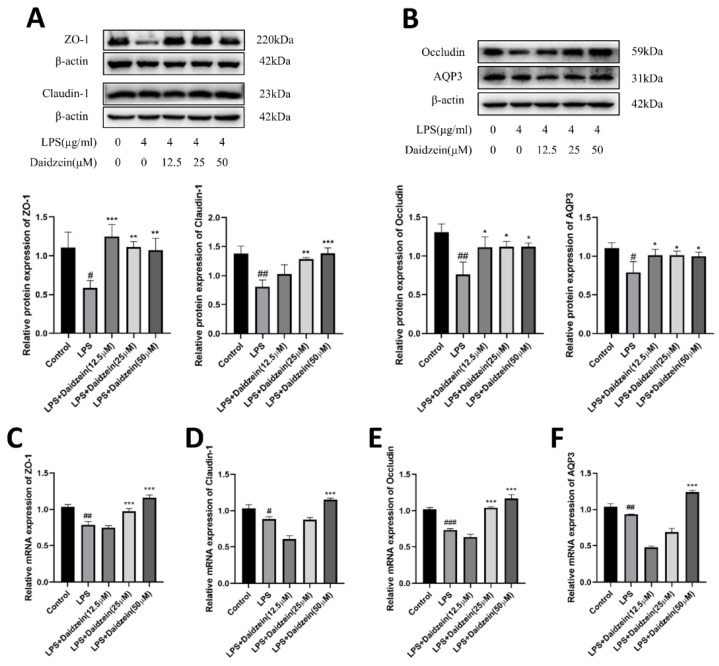
Daidzein increased lipopolysaccharide (LPS)-induced downregulated expression of tight junction and AQP3 proteins and mRNA. (**A**) The protein expression levels of ZO-1 and occludin and protein quantitative analysis. (**B**) The protein expression levels of claudin-1 and AQP3 and protein quantitative analysis. (**C**–**F**) The mRNA expression levels of ZO-1, occludin, claudin-1, and AQP3 (mean ± SD, *n* = 3), * *p* < 0.05, ** *p* < 0.01, and *** *p* < 0.001 versus LPS-treated alone. # *p* < 0.05, ## *p* < 0.01, and ### *p* < 0.001 versus control.

The expressions of ZO-1, occludin, claudin-1, and AQP3 proteins were measured by Western blotting to assess whether daidzein possessed positive effects on LPS-induced intestinal epithelial barrier injury. As shown in Figure 3A,B, ZO-1, occludin, claudin-1, and AQP3 protein expression levels in Caco-2 cells both decreased (*p* < 0.05, *p* < 0.01) in the LPS-treated group, whereas daidzein (12.5, 25, or 50 μM) treatment markedly increased (*p* < 0.05) the ZO-1, occludin, and AQP3 proteins levels, and daidzein (25 or 50 μM) treatment upregulated (*p* < 0.01) the claudin-1 protein level. Similarly, the mRNA expressions of ZO-1, occludin, claudin-1, and AQP3, determined by qRT-PCR analysis, declined in the LPS-treated group (Figure 3C–F, *p* < 0.05). Compared with the LPS-treated group, the 25 μM daidzein-treated group statistically increased the ZO-1 and occludin mRNA expression (*p* < 0.01), and the 50 μM daidzein-treated group noticeably increased the mRNA expression of ZO-1, occludin, claudin-1, and AQP3 (*p* < 0.05). Above all, these outcomes show that 50 μM daidzein could increase the expression of TJ and AQP3 proteins and mRNA expression, and that the low concentration of daidzein could mainly increase the expression of TJ and AQP3 proteins.

**Figure 4:**
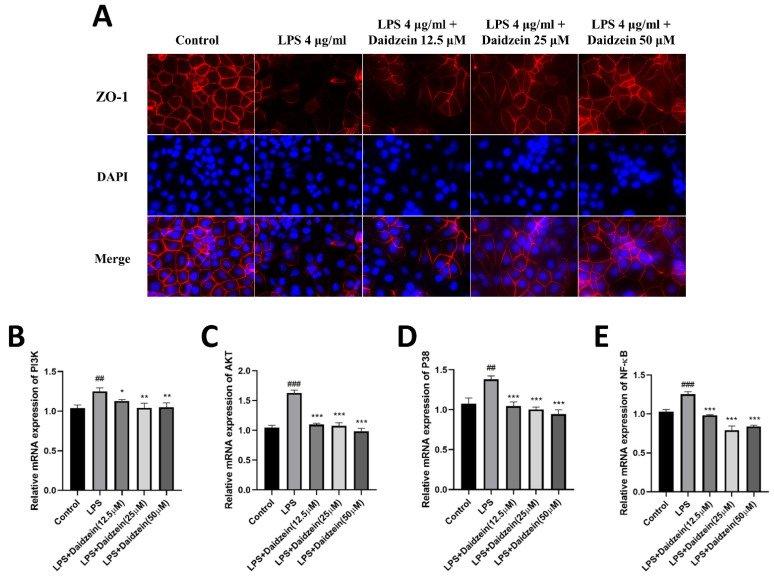
Daidzein ameliorated the tight junction of the ZO-1 protein and suppressed the gene expression of the PI3K/AKT and P38 pathways. (**A**) The intercellular junction of the ZO-1 protein. The red signal indicates ZO-1, and the blue one indicates nuclei. (**B**,**C**) The mRNA expression levels of the PI3K/AKT pathways. (**D**) The mRNA expression levels of the P38 pathways. (**E**) The mRNA expression levels of p-NF-κB and NF-κB (mean ± SD, *n* = 3), * *p* < 0.05, ** *p* < 0.01, and *** *p* < 0.001 versus LPS-treated alone. ## *p* < 0.01, and ### *p* < 0.001 versus control.

To further confirm that daidzein protected the Caco-2 cell monolayers against the LPS-induced degradation of TJ proteins, we used immunofluorescence (IF) assays to determine the intercellular junction of the ZO-1 protein in Caco-2 cells. The intercellular junction of the ZO-1 protein was destroyed by 4 μg/mL LPS, whereas daidzein could maintain its integrity (Figure 4A). The IF staining results were consistent with the Western blotting and qRT-PCR results, and suggested a protective influence of daidzein on TJ proteins.

### 2.4. Daidzein Inhibited LPS-Induced PI3K/AKT and P38 Pathway Activation in Caco-2 Cells

**Figure 5:**
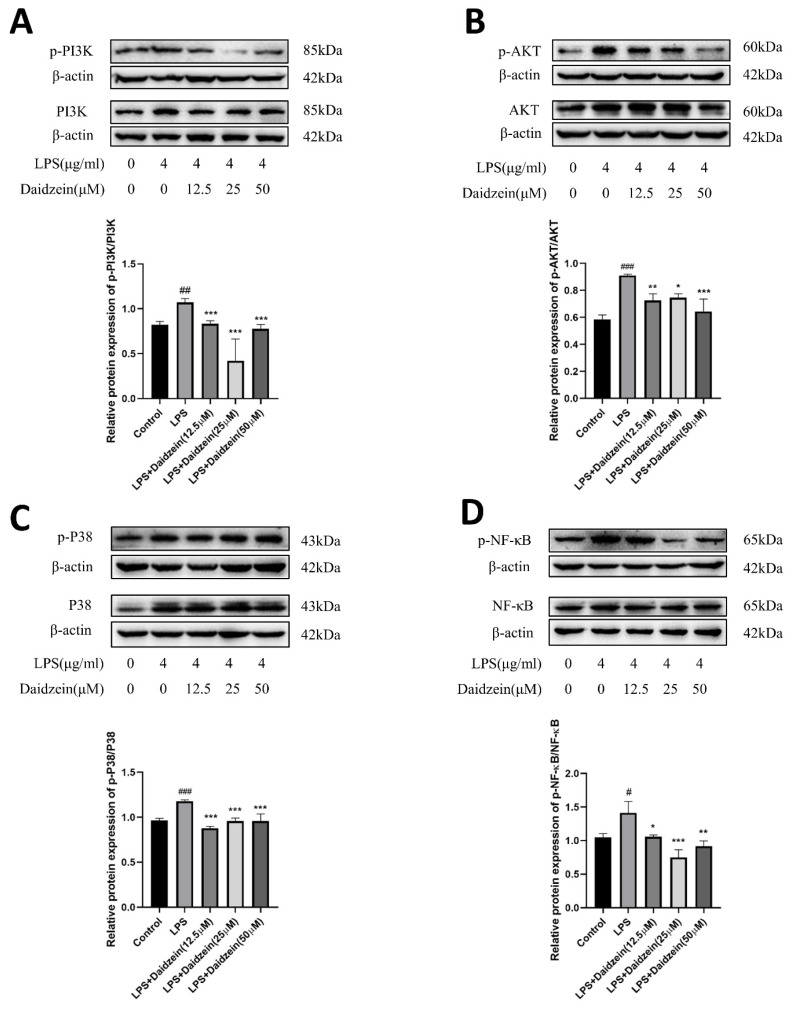
Daidzein inhibited LPS-induced upregulated protein expression of the PI3K/AKT and P38 pathways. (**A**,**B**) The protein expression levels of the PI3K/AKT pathways. (**C**) The protein expression levels of the P38 pathway. (**D**) The protein expression levels of p-NF-κB and NF-κB (mean ± SD, *n* = 3), * *p* < 0.05, ** *p* < 0.01, and *** *p* < 0.001 versus LPS-treated alone. # *p* < 0.05, ## *p* < 0.01, and ### *p* <0.001 versus control.

The levels of the PI3K/AKT and P38 pathway-related genes and proteins were detected by qRT-PCR and Western blot, respectively. As displayed in Figure 4B–E, LPS provoked the gene expression of PI3K, AKT, NF-κBp65, and P38 compared with the control groups, whereas treatment with daidzein clearly reversed the aforementioned impacts (*p* < 0.05). In the protein expression analysis, we examined the levels of p-PI3K, p-AKT, p-P38, and p-NF-κB in Caco-2 cells treated with daidzein (12.5–50 μM) for 24 h by Western blotting (Figure 5A–D). Relative to the LPS-treated group, treatment with daidzein clearly reduced phosphorylated PI3K, AKT, and NF-κBp65 (*p* < 0.05). These results indicate that daidzein may promote TJ and AQP3 protein expression by inhibiting the activation of the PI3K/AKT and P38 pathways.

### 2.5. Daidzein Improved LPS-Induced Downregulation Expression of TJ and AQP3 Proteins by Inhibiting PI3K/AKT and P38 Pathways

**Figure 6:**
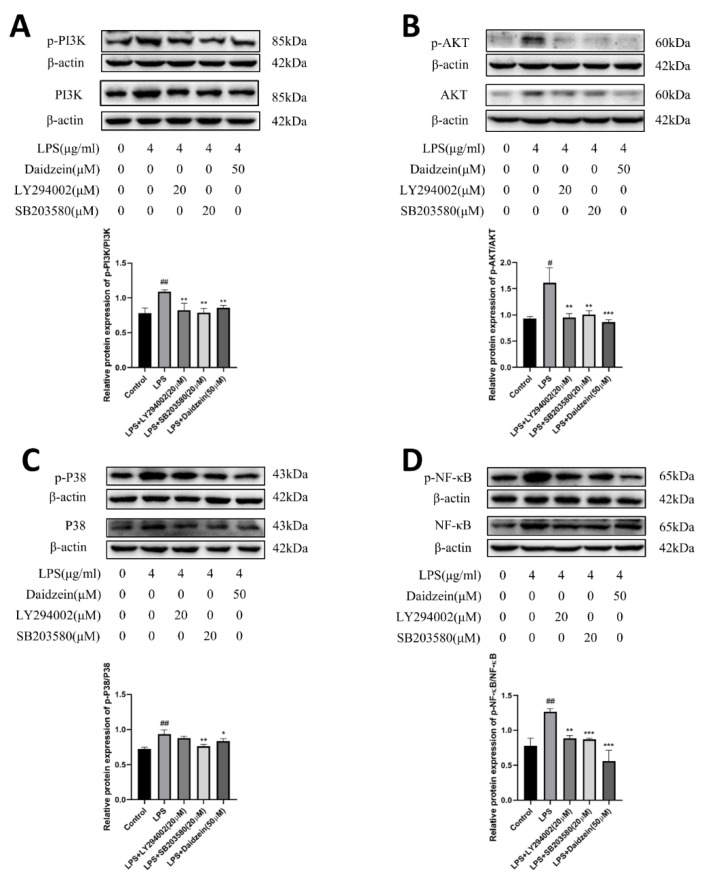
Daidzein suppressed the LPS-induced effects through the PI3K/AKT and P38 pathways. (**A**,**B**) The protein expression levels of the PI3K/AKT pathways. (**C**) The protein expression levels of the P38 pathway. (**D**) The protein expression levels of p-NF-κB and NF-κB (mean ± SD, *n* = 3), * *p* < 0.05, ** *p* < 0.01, and *** *p* < 0.001 versus LPS-treated alone. # *p* < 0.05, and ## *p* < 0.01 versus control.

**Figure 7:**
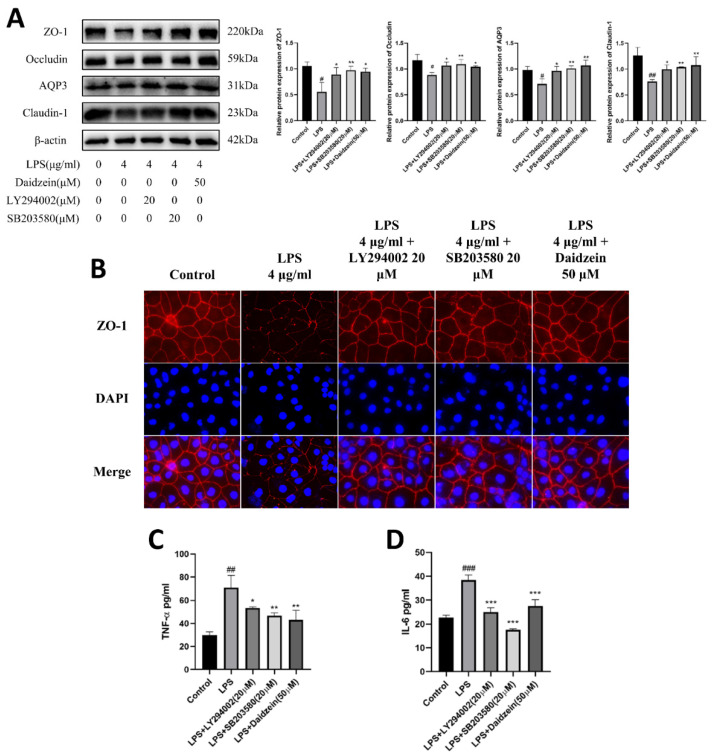
Daidzein improved the LPS-induced downregulation expression of tight junctions and AQP3 proteins and inflammation by inhibiting the PI3K/AKT and P38 pathways. (**A**) The protein expression levels of ZO-1, occludin, claudin-1, and AQP3 and protein quantitative analysis. (**B**) The tight junction of the ZO-1 protein. The red signal indicates ZO-1, and the blue one indicates the nuclei. (**C**) The level of TNF-α. (**D**) The level of IL-6 (mean ± SD, *n* = 3), * *p* < 0.05, ** *p* < 0.01, and *** *p* < 0.001 versus LPS-treated alone. # *p* < 0.05, ## *p* < 0.01, and ### *p* < 0.001 versus control.

The aim was to investigate whether daidzein regulated TJ and AQP3 protein expressions via the PI3K/Akt and P38 pathways. Caco-2 cells were incubated with LPS (4 μg/mL) plus PI3K inhibitor (LY294002, 20 μM) or P38 inhibitor (SB203580, 20 μM) for 24 h to verify the inhibition of the PI3K/AKT and P38 pathways. LY294002 reversed the increased phosphorylation of the PI3K/AKT pathways, and SB203580 markedly reduced the phosphorylation of the PI3K/AKT and P38 pathways in the LPS-treated Caco-2 cells (*p* < 0.05, Figure 6A–D). Moreover, relative to the LPS-treated group, ZO-1, occludin, claudin-1, and AQP3 expression were all increased (*p* < 0.05) in the LY294002 or SB203580 treatment groups, as with the daidzein-treated group (Figure 7A). Furthermore, the IF staining results were consistent with the results of the daidzein treatment group (Figure 7B), which manifested that daidzein could weaken LPS-induced diminishment of TJ and AQP3 protein expressions by inhibiting the PI3K/AKT and P38 pathways.

### 2.6. Daidzein Suppressed LPS-Induced Inflammation through PI3K/AKT and P38 Pathways

The roles of the PI3K/AKT and P38 pathways in the anti-inflammatory effect of daidzein were further explored. LPS treatment alone significantly increased (*p* < 0.01) the secretion of TNF-α and IL-6 in the supernatant of the Caco-2 cell culture, whereas treatment with 50 μM daidzein significantly lessened (*p* < 0.01) TNF-α and IL-6 secretion caused by LPS. In addition, treatment with daidzein was found to have similar effects as treatment with LY294002 (20 μM) or SB203580 (20 μM), with both significantly different compared with the LPS-treated group (*p* < 0.05, Figure 7C,D). In conclusion, daidzein inhibited inflammation by disrupting the PI3K/AKT and P38 pathways in the Caco-2 cells.

## 3. Discussion

In this study, we used a LPS-induced Caco-2 cell monolayer injury model to explore how daidzein ameliorated intestinal epithelial barrier injury. In the LPS-induced Caco-2 cells, we found that daidzein could improve the disruption of the Caco-2 cell monolayers’ integrity, upregulate the expressions of ZO-1, occludin, claudin-1, and AQP3 proteins, and reduce the level of TNF-α and IL-6 as well as inhibit the PI3K/AKT and P38 pathway related protein expressions. In addition, we used the PI3K/AKT inhibitor LY294002 and P38 inhibitor SB203580 to further demonstrate that the mechanism of daidzein might be closely related to inhibition of the PI3K/AKT and P38 pathways via the PI3K/AKT and P38 pathways.

Intestinal epithelium, a single-cell layer, provides an important barrier against bacterial endotoxin translocation. TJ proteins are apical components of the intestinal epithelial barrier and maintains the integrity of the barrier [23]. AQP3 plays a critical role in regulating the water content in the human colon, which is responsible for the normal dehydration of fecal matter [24]. Moreover, it has been reported that with the knockdown of AQP3, the expression of TJ complexes is decreased and colonic epithelial permeability is increased [16]. This makes daidzein increased AQP3 protein an effective strategy for intestinal epithelial barrier protection. In this study, we found how daidzein regulated TJ and AQP3 proteins to exert the effect of protecting the intestinal epithelial barrier. We demonstrated that LPS significantly reduced transepithelial electrical resistance (TEER) and increased paracellular permeability in the Caco-2 cell monolayers, while daidzein treatment reversed the above-mentioned impacts of LPS. Moreover, we found that daidzein upregulated the protein level of ZO-1, occludin, claudin-1, and AQP3, and maintained the intercellular junction of ZO-1. Therefore, we speculated that daidzein protects Caco-2 from LPS-induced injury and preserves the epithelium barrier function through enhancing TJ and AQP3 protein expression.

Intestinal epithelial barrier injury caused by various pathogen infections is often related to inflammatory damage [2,3]. High levels of inflammatory factors such as IL-6 have been shown to be associated with persistent diarrhea and adverse clinical outcomes [25]. It has been reported that TNF-α or IL-6 was related to dysfunction of the intestinal barrier and apoptosis of intestinal epithelial cells [26,27,28]. NF-κB is involved in the production of pro-inflammatory cytokines and the regulation of various inflammatory signaling pathways, which could increase the level of pro-inflammatory factors such as TNF-α and IL-6 [29,30]. In this study, the 50 μM daidzein treatment group showed significantly decreased secretion levels of IL-6 and TNF-α from the Caco-2 cells as well as the expression of NF-κB. These outcomes suggest that daidzein delivers a protective effect on the intestinal epithelial barrier, which may be associated with the suppression of the cellular inflammatory response.

**Figure 8:**
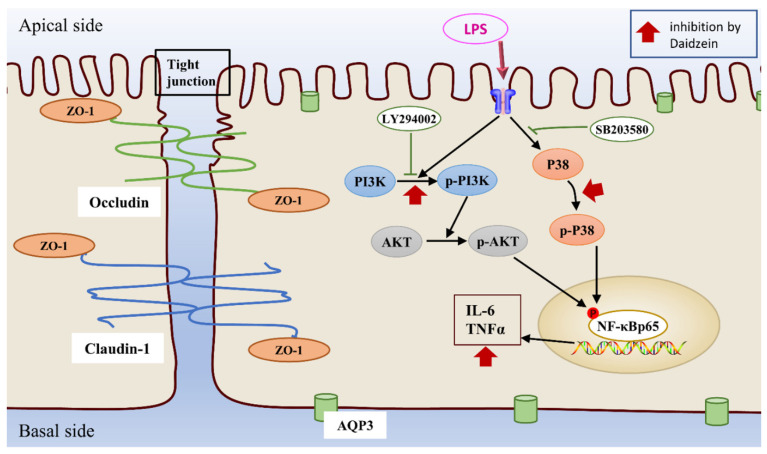
The possible mechanism of daidzein in the treatment of LPS-induced intestinal epithelial barrier injury.

The P38 MAPK and PI3K/AKT cascade are both essential signaling pathways in cell growth, survival, immunity, and apoptosis when inflammation occurs [31,32]. PI3K/AKT signaling can inhibit the phosphorylation of IκBα activating the NF-κB signaling pathway, while P38 MAPK signaling is involved in various inflammatory factor expressions [33,34]. The literature has shown that P38 MAPK activation inhibits the effect of the PI3K/AKT pathway, which then reduces IL-8-induced neutrophil chemotaxis [35]. Meanwhile, many studies have demonstrated that PI3K/AKT can inhibit the P38 MAPK pathway and have opposing effects on apoptosis [36,37]. However, most chemokines mediate the migration of neutrophils and induce inflammatory responses at the site of infection through these two typical pathways [35]. In this study, we quantified the PI3K/AKT and P38 signaling pathways to reveal the anti-inflammatory and mechanism of daidzein in protecting the intestinal epithelial barrier. Daidzein was able to decrease the phosphorylation of PI3K, AKT, and P38 as well as reduce the phosphorylated NF-κB and the level of TNF-α or IL-6, which partially supports the hypothesis that daidzein weakens inflammation and reduces disruption of the barrier by inhibiting LPS-induced activation of the PI3K/AKT and P38 pathways. To further investigate whether daidzein regulated TJ and AQP3 protein expression via the PI3K/AKT and P38 pathways, we added pathway inhibitors to the cells. LY294002 is a pan-PI3K inhibitor, which was proven to inhibit LPS-induced acute hepatitis injury in a murine model and also reduced TNF-α and IL-6 expression [38]. SB203580 is a P38MAPK pathway inhibitor, which can reverse LPS-induced lung injury by mediating the P38MAPK/NF-κB pathway [39]. Similar outcomes were observed in our study, LY294002 and SB203580 diminished the expression of PI3K, AKT, P38, NF-κB, TNF-α, and IL-6 in LPS-stimulated Caco-2 cells. Furthermore, LY294002 and SB203580 upregulated ZO-1, occludin, claudin-1, and AQP3 expression. Our research revealed that daidzein might play a protective role in intestinal epithelial barrier by reducing the inflammatory response via inhibiting the PI3K/AKT and P38 signaling pathways (Figure 8).

Taken together, this research demonstrated that daidzein exhibited a protective effect on LPS-induced intestinal epithelial barrier injury through the modulation of the PI3K/AKT and P38 pathways. Daidzein promotes ZO-1, occludin, claudin-1, and AQP3 expression by the PI3K/AKT and P38 pathway suppression of NF-κB, TNF-α, and IL-6 expression, which, in turn, reverses LPS-induced injury. These findings clarify the molecular mechanism by which daidzein exerted protective effects on the barrier. The results may provide scientific support for daidzein as a dietary supplementation or drug to prevent or cure diarrhea. However, the further mechanism of anti-diarrhea in vivo is necessary to be explored.

## 4. Materials and Methods

### 4.1. Reagents

Daidzein (CAS. 486-66-8, purity ≥ 98%) originated from the National Institute for the Control of Pharmaceutical and Biological Products (Beijing, China). DAPI, with an antifade mounting medium, was obtained from Beyotime (Shanghai, China). Fluorescein isothiocyanate dextran of molecular mass 4 kDa (FITC-FD4) was purchased from Sigma Aldrich (St. Louis, MO, USA). Lipopolysaccharide (LPS) from *E. coli* 0111: the B4 strain was purchased from Invivogen (San Diego, CA, USA). The PI3K/AKT pathway inhibitor (LY294002) and P38 pathway inhibitor (SB203580) were purchased from Selleck (Houston, TX, USA).

Primary antibodies against anti-rabbit AQP3 (Cat# AF5222), NF-κBp65 (Cat# 10745-1-AP), P38 (Cat# AF6456), p-P38 (Cat# AF4001), β-actin (Cat# AF7018), and goat anti-rabbit flour488 (Cat# S0018) were purchased from Affinity Biosciences (Cincinnati, OH, USA). Primary antibodies against anti-rabbit ZO-1 (Cat# 8193T), PI3K (Cat# 4257T), p-PI3K (Cat# 4228T), AKT (Cat# 4691T), p-AKT (Cat# 4060T), p-NF-κBp65 (Cat# 3033T), HRP-linked antibody (Cat# 7074S), and anti-rabbit Alexa Flour^®^ 594 (Cat# 8889S) were purchased from Cell Signaling Technology (Danvers, MA, USA). Occludin (Cat# 27260-1-AP) and claudin-1 (Cat# 13050-1-AP) were purchased from Proteintech (Chicago, IL, USA).

### 4.2. Cell Culture and Solution Preparation

Caco-2, the human epithelial colorectal adenocarcinoma cell line, was purchased from Jennio (Guangzhou, China), cultured in complete minimum essential medium (MEM, Gibco, NY, USA) with 10% fetal bovine serum (Gibco), and cultivated in a humidified incubator of 95% air and 5% CO_2_ at 37 °C. Experiments were performed utilizing 20–50 generations of cells.

Daidzein, LY294002, and SB203580 were respectively dissolved into dimethyl sulfoxide (DMSO) with the final concentration <0.1% in order to have no significant effects on the viability of the Caco-2 cells.

### 4.3. Cell Viability Assays

Caco-2 cells (1 × 10^3^ cells per well) were seeded in 96-well plates and then treated with LPS (5–20 μg/mL), daidzein (12.5–200 μM), daidzein (12.5–50 μM) plus 4 μg/mL LPS, or LY294002 (20 μM) plus 4 μg/mL LPS, or SB203580 (20 μM) plus 4 μg/mL LPS for 24 h. After treatment, the Caco-2 cells were treated with the Cell Counting Kit-8 (CCK-8, Gibco) for 1 h, and then their absorbance was measured at 450 nm using Microplate Reader (BioTek, Winooski, VT, USA).

### 4.4. Intestinal Epithelial Barrier Function Measurement

Transepithelial electrical resistance (TEER) is utilized as an indicator of the monolayer fusion and integrity. Caco-2 cells (1.5 × 10^4^ cells per well) were placed in Transwell plates (Corning, MA, USA) with polyester membranes (0.33 cm^2^, 0.4 μm pore) and monitored until the TEER values had achieved >300 Ω • cm^2^, demonstrating a tight monolayer. After that, cells were treated with LPS (1–4 μg/mL), or LPS (4 μg/mL) plus daidzein (12.5–50 μM) for 24 h. TEER was measured using the Millicell^®^ ERS-2 voltmeter (Merck Millipore, Darmstadt, Germany) after washing the cells twice with PBS. TEER was calculated as: TEER (Ω • cm^2^) = (Cell resistance − Cell-free resistance) Ω × 0.33 cm^2^.

The flux of FITC-FD4 was measured to evaluate the permeability of the human intestinal Caco-2 cell monolayers. After testing TEER, 100 μg/mL of FITC-FD4 was added to the apical sides and incubated for 2 h. Later, the basolateral medium was collected into 96-well plates and the fluorescence intensity was determined using a Fluorescence Microplate Reader (BioTek, VT, USA) with excitation at 492 nm and emission at 520 nm.

### 4.5. Enzyme-Linked Immunosorbent Assays (ELISA)

The level of interleukin-6 (IL-6) and tumor necrosis factor α (TNF-α) in the supernatant of different administration cell groups, as described above, were determined by using IL-6 Enzyme-Linked Immunosorbent Assay (ELISA) Kits (Cat# MM0049H2) and TNF-α ELISA Kits (Cat# MM0122H2) (Sorfa Life, Huzhou, China), respectively.

### 4.6. Quantitative Real-Time Polymerase Chain Reaction (qRT-PCR) Analysis

The total RNA was separated by RNA extraction solution according to the manufacturer’s manual and the concentration determined with a Nanodrop 2000 (Nano-Drop Technologies, DE, USA). Later, RNA was reverse transcribed by a First Strand cDNA Synthesis Kit (Servicebio, Wuhan, China). RT-PCR was conducted using 2 × SYBR Green qPCR Master Mix. The sequences of the primers are presented in Table 1.

### 4.7. Immunofluorescent (IF) Analysis

Caco-2 cells (2 × 10^5^ cells per well) were cultured on 6-well plates and subjected to LPS (4 μg/mL), LPS (4 μg/mL) plus daidzein (12.5, 25, 50 μM), or plus LY294002 (20 μM), or plus SB203580 (20 μM). After administration for 24 h, cells were immersed in 4% paraformaldehyde for 10 min, followed by blocking with 5% BSA for 1 h and incubating with primary antibody (ZO-1 (1:1000)) at 4 °C overnight. Then, the cells were incubated with anti-rabbit Alexa Flour^®^ 594 or goat anti-rabbit flour488 for 1 h in the dark, and counterstained with antifade mounting medium with DAPI and visualized with an LSM 800 microscope (Zeiss, Oberkochen, Germany).

### 4.8. Western Blotting Analysis

The whole protein was extracted from Caco-2 cells with different administration methods. Briefly, cells were lysed in RIPA lysis buffer at 4 °C and centrifuged for 15 min at 4 °C, followed by supernatant separation and measured the whole protein concentration by a BCA Protein Assay Kit (Keygen Biotech, Jiangsu, China). Cell lysate was separated by 12% SDS-PAGE and the proteins were transferred onto a 0.45 μm polyvinylidene fluoride membrane (Merck, Darmstadt, Germany). Membranes were incubated with the primary antibody (ZO-1 (1:1000), occludin (1:1000), claudin-1 (1:1000), AQP3 (1:1000), NF-κBp65 (1:1000), p-NF-κBp65 (1:1000), PI3K (1:1000), p-PI3K (1:1000), P38 (1:1000), p-P38 (1:1000), and β-actin (1:5000)) at 4 °C overnight. Finally, membranes were incubated with a HRP-linked secondary antibody for 1 h and photographed by a Tanon 5200s (Tanon, Shanghai, China).

### 4.9. Statistical Analysis

GraphPad Prism 7.0 software was performed for the data analysis. All results were displayed as mean ± SD and repeated triplicate times. The significance of difference between groups was estimated by one-way analysis of variance, followed by Tukey’s test. A *p*-value < 0.05 represents the statistical significance.

## Figures and Tables

**Table 1:** The following mRNA primer sequences were used for reverse transcription-quantitative PCR.

Gene	Upstream Primer Sequence	Downstream Primer Sequence
ZO-1	AAACAAACCCAGCATCATCAAC	TGTGCCCTGGGTGACTAACG
Occludin	TTCCTATAAATCCACGCCGG	TGTCTCAAAGTTACCACCGCTG
Claudin-1	GCCAGGTACGAATTTGGTCAG	TTGGTGTTGGGTAAGAGGTTGT
AQP3	GACCCTCATCCTGGTGATGTTT	GCCCAGAGTGACAGCAAAGC
PI3K	ACTGCCGAGAGATTTTCCCAC	TCACTCATCTGTCGCAGGCA
AKT	TACTCTTTCCAGACCCACGACC	CCCGGTACACCACGTTCTTCT
P38	GCTCTCCAGACCATTTCAGTCC	CATGAGATGGGTCACCAGATACAC
NF-κB	TCCCATCTTTGACAATCGTGC	AGCCTGGTCCCGTGAAATAC
Gapdh	GGAAGCTTGTCATCAATGGAAATC	TGATGACCCTTTTGGCTCCC

## Data Availability

All datasets generated in this study are included in the article.

## Abbreviations

AQP3, aquaporin 3; Caco-2, the human epithelial colorectal adenocarcinoma cell line; ELISA, enzyme-linked immunosorbent assay; IL-6, interleukin-6; LPS, lipopolysaccharide; NF-κB, nuclear factor-Κb; PI3K, phosphoinositide-3 kinase; TJ, tight junction; TNF-α, tumor necrosis factor-α; ZO, zonula occludens.
